# Activation of the HNRNPA2B1/*miR-93-5p*/FRMD6 axis facilitates prostate cancer progression in an m6A-dependent manner

**DOI:** 10.7150/jca.83863

**Published:** 2023-05-08

**Authors:** Menghao Sun, Yuanhao Shen, Gaozhen Jia, Zheng Deng, Fei Shi, Yifeng Jing, Shujie Xia

**Affiliations:** 1 Clinical Medical Center of Urology, Shanghai General Hospital, Shanghai Jiao Tong University School of Medicine, Shanghai, China.; 2 Institute of Urology, Shanghai Jiao Tong University, Shanghai, China.; 3 Cancer Center, Shanghai General Hospital, Shanghai Jiao Tong University School of Medicine, Shanghai, China.

**Keywords:** HNRNPA2B1, Prostate cancer, m6A, *miR-93-5p*, FRMD6

## Abstract

It is becoming increasingly clear that N6-methyladenosine (m6A) plays a key role in post-transcriptional modification of eukaryotic RNAs in cancer. The regulatory mechanism of m6A modifications in prostate cancer is still not completely elucidated. Heterogeneous nuclear ribonucleoprotein A2/B1 (HNRNPA2B1), an m6A reader, has been revealed to function as an oncogenic RNA-binding protein. However, its contribution to prostate cancer progression remains poorly understood. Here, we found that HNRNPA2B1 was highly overexpressed and correlated with a poor prognosis in prostate cancer. *In vitro* and* in vivo* functional experiments demonstrated that *HNRNPA2B1* knockout impaired proliferation and metastasis of prostate cancer. Mechanistic studies indicated that HNRNPA2B1 interacted with primary *miRNA-93* and promoted its processing by recruiting DiGeorge syndrome critical region gene 8 (DGCR8), a key subunit of the Microprocessor complex, in an METTL3-dependent mechanism, while *HNRNPA2B1* knockout significantly restored *miR-93-5p* levels. HNRNPA2B1/*miR-93-5p* downregulated FERM domain-containing protein 6 (FRMD6), a cancer suppressor, and enhanced proliferation and metastasis in prostate cancer. In conclusion, our findings identified a novel oncogenic axis, HNRNPA2B1/*miR-93-5p*/FRMD6, that stimulates prostate cancer progression via an m6A-dependent manner.

## Introduction

Globally, 1.4 million new cases of prostate cancer (PCa) were diagnosed in 2020, making it the fourth most common cancer diagnosis 1. Multiple treatment strategies for prostate cancer are available, including surgery, radiotherapy, chemotherapy, and hormonal therapy 2. Among these management options, radical prostatectomy can cure localized prostate cancer, while for patients with advanced stages, androgen deprivation therapy is the primary treatment 3. However, once the tumor breaches the surgical capsule of the prostate and metastasizes to remote organs, patients lose the opportunity for curative surgery. Based on many successful studies, tumorigenesis and progression of prostate cancer are complex processes involving not only abnormal genetic changes but also epigenetic disorders 4, 5. Therefore, exploring the mechanisms of prostate cancer development and subsequently identifying new therapeutic targets remains a great challenge.

Epigenetic modifications associated with cancer development can happen at many levels, such as methylation of DNA and RNA, histone modification, and chromatin remodeling, among which DNA and RNA methylation are of particular importance 6. In eukaryotes, N6-methyladenosine (m6A) RNA methylation, as a highly abundant RNA modification, has been identified as a key post-transcriptional modification, mediating gene expression by affecting splicing regulation, RNA stability, processing of primary microRNA (pri-miRNA), and translation of mRNA. As a dynamic process, RNA m6A can be imprinted by “writers”, eliminated by “erasers”, and functionally identified by “readers” 7, 8. In recent years, a surge in evidence supported an important regulatory role for m6A in tumorigenesis and development 9. Chen et al. identified that high levels of total m6A modifications were markedly prevalent in PCa 10. Further, Li et al. verified that YTHDF2 degraded m6A-modified mRNAs in a METTL3-dependent way, which promoted tumor development and metastasis 11. However, there is still a great deal of mystery surrounding the mechanism by which m6A modifications regulate prostate cancer progression.

Therefore, we systematically analyzed transcriptome data and clinicopathological information of patients from prostate adenocarcinoma (PRAD) cohort of The Cancer Genome Atlas (TCGA) for 18 m6A-related genes. After a thorough analysis and screening, we found that HNRNPA2B1 appeared to play a critical role in PCa. HNRNPA2B1 has been reported as a nuclear m6A reader that positively regulated the processing of a subset of METTL3-dependent pri-miRNAs, by binding their m6A loci and interacting with microprocessor protein DGCR8 12. Moreover, increased expression of HNRNPA2B1 was observed in various malignancies and its upregulation affected malignant phenotypes of cancer cells by modulating a wide range of downstream genes 13. However, as far as we know, a role of HNRNPA2B1 as an m6A reader has not yet been reported in prostate cancer.

In the present study, we focused on the m6A-related function of HNRNPA2B1 in prostate cancer. We found that HNRNPA2B1 was highly expressed in prostate cancer and related to adverse clinicopathological features and poor prognosis. Moreover, by recruiting the microprocessor protein DGCR8, HNRNPA2B1 positively modulated the processing of *pri-miR-93* via an m6A-depenent way and subsequently inhibiting its target gene *FRMD6*, a cancer suppressor gene.

## Materials and Methods

### Bioinformatics data collection

Gene expression data and corresponding clinicopathological information of prostate cancer patients were acquired from TCGA-Prostate Adenocarcinoma (PRAD) and Gene Expression Omnibus (GEO) databases. TCGA-PRAD database includes 52 normal prostate samples and 499 prostatic carcinoma tissues. GSE3325, GSE6919, GSE29079, and GSE94767 provided mRNA expression data of prostatic samples from PCa patients. Additionally, GSE14857, GSE17317, GSE21036, GSE36802, and GSE60117 provided miRNA expression data of prostate cancer patients. Before further analysis, samples without reliable clinical information were excluded.

### Bioinformatics analysis

Our study analyzed the expression data of 18 selected genes from TCGA-PRAD database using the R package “limma”. In order to visualize the expression data, the R packages "ComplexHeatmap" and "ggplot2" were used to create a heatmap and bar charts. The expression levels of target genes and miRNAs from the GEO database were analyzed with the R package “DESeq2”. Next, we assessed the overall survival (OS) time and progression-free survival (PFS) time associated with the 18 genes from TCGA-PRAD database using univariate Cox regression analysis. The forest plots were made with the R packages “survival” and “survminer”. Then, we used univariate Cox regression analysis to explore the independent prognostic value of clinical features of the target genes and miRNAs from TCGA-PRAD database and GSE series. We analyzed features including TMN stages, Gleason scores, and prostate-specific antigen levels. Scatter figures were plotted to show the comparisons using the R package “ggplot2”. In order to determine the relevance of gene expression levels between miRNAs and selected genes, Spearman correlation analysis was applied.

### PCa tissue specimen and cell lines

Prostate cancer specimens and paired adjacent non-tumor tissues were acquired from patients with diagnosed prostate adenocarcinoma at Shanghai General Hospital (Shanghai, China) between 2018 and 2020. Patients included had not received radiotherapy, hormonal therapy, chemotherapy, or any other non-surgical treatments before radical prostatectomy. Ethical approval was obtained from Shanghai General Hospital for the use of clinical samples in this study. Informed consent forms were signed by all patients involved. Clinicopathological characteristics of PCa patients are listed in Table S1.

Two non-malignant human prostate cell line (RWPE-1 and BPH-1) and four malignant prostate cell lines (22RV1, DU145, PC3, and LNCaP) were obtained from the Cell Bank of the Type Culture Collection of Chinese Academy of Sciences (Shanghai, China). Cell culturing conditions of above cell lines were the same as a previous study 14.

### Immunohistochemistry

To conduct immunohistochemical analysis on collected tissues, paraffin sections were prepared. After dewaxing and rehydrating, sections underwent antigen retrieval with a citric acid buffer. The IHC staining was carried out as previously described 14. To quantify the protein levels of each sample, ImageJ was used to calculate the integrated optical density per stained area (IOD/area, AOD). The information of all primary antibodies used in this study are presented in Table S2.

### Western blotting

Prepared cells for Western blotting assays were lysed using radioimmunoprecipitation assay (RIPA) lysis buffer (Sigma-Aldrich, USA) supplemented with protease and phosphatase inhibitors (ThermoFisher Scientific, USA), scraped, and collected in 1.5-mL Eppendorf tubes. After examining the concentration of each protein samples using a BCA protein quantitation kit (Vazyme, China), Western blotting assays were performed as previously described 15. The information of all primary antibodies used in this study are presented in Table S2.

### *HNRNPA2B1* knockout using a CRISPR-Cas9 system

An sgRNA cloned lenti-CRIPSR-V2 vector and control vector, as well as psPAX2 and Pmd2.G, were co-transfected into HEK293T cells using Lipofectamine 3000 (Invitrogen, USA). A concentrated virus supernatant supplemented with polybrene was used for lentivirus infection. Then, 1.5 μg/mL puromycin was used for cell selection until stable *HNRNPA2B1* knockout cell lines were established. Sequences of the sgRNAs are shown in Table S3.

### Cell proliferation and colony formation assay

A cell counting kit-8 (CCK-8; NCM, China) was used to examine cell proliferation, following the procedures as described in our previous paper 15.

For colony formation assays were performed as described previously 16.

### Cell migration and invasion assay

To assess migration and invasion of cells, trans-well chamber assays were employed. Procedures of this experiment were in accordance with a previous study 16.

### Animal experiments

Male four-week-old BALB/C nude mice were inoculated subcutaneously in the right axilla with 1×10^6^ HNRNPA2B1 knockout or negative control DU145 cells. Xenograft tumor growth was recorded weekly by measuring the width (W) and length (L) of the tumor with vernier calipers and calculating the tumor volume (V, mm^3^) using the formula V = W^2^ × L × 0.52. Xenograft tumors were harvested and weighed five weeks after cell injection.

To mimic the systemic metastasis of prostate cancer, we constructed a metastasis model. Specifically, 3×10^5^ PC3-control knockout or PC3-*HNRNPA2B1* knockout cells transfected with a luciferase-expressing lentiviral vector were slowly inoculated into the left ventricle of the mice by intracardiac injection using a 1-mL tuberculin syringe, and the entire procedure was performed under the guidance of an animal ultrasound imaging system. During needling, a pulsatile bright red arterial blood flow was considered as a sign of left ventricle administration, according to a previous study 17. All mice were imaged for luciferase activity weekly after the PCa cell administration to monitor tumor metastasis using an IVIS live animal imaging system. Above animal experiments were approved by the Scientific Investigation Board of Shanghai General Hospital and implemented following the US Public Health Service Policy on Humane Care and Use of Laboratory Animals.

### Plasmid construction and cell transfection

METTL3-targeting siRNAs, *miR-93-5p* mimics, and *miR-93-5p* inhibitors were obtained from RiboBio (China) and transfected into PCa cells using a transfection reagent, Lipofectamine 3000 (Invitrogen, USA), following the manual provided by the manufacturer. The lentiviral vector containing the *FRMD6* sequence and negative control were constructed by RiboBio (China) to infect DU145 and PC3 cells and obtain stable *FRMD6*-overexpressing cell lines after screening with puromycin. All the information for the above sequences is listed in Table S4.

### RNA isolation and qRT-PCR

In accordance with the previous description, the specific procedures were carried out 18. All the primer sequences are listed in Table S5.

### RNA immunoprecipitation (RIP)

All RIP assays were performed with a Magna RIP kit from Millipore according to the instruction provided by the manufacturer. Briefly, cells were completely lysed in lysis buffer and processed for immunoprecipitation. The purified RNAs were analyzed by qRT-PCR, and the level of the target genes was normalized to the input. Antibodies and primers used in RIP assays are listed in Table S2 and Table S5, respectively.

### Co-immunoprecipitation assay

Co-immunoprecipitations (Co-IP) assays in this study were conducted with a Co-IP kit (BerSinBio, China) strictly following the instructions from the manufacturer. Briefly, cells were harvested and lysed. Next, 10% of the lysate was used as the input, while two volumes, each containing 40% of the lysate, were incubated with IgG or primary antibodies at 4°C overnight, and then incubated with magnetic beads at room temperature. The proteins eluted from the beads were analyzed by western blotting. Antibodies used in the Co-IP assay are listed in Table S2.

### Dual-luciferase reporter assay

Human *FRMD6* 3'UTR region and its sequence with a mutation of the *miR-93-5p* seed sequence were inserted into a firefly/Renilla luciferase reporter vector (pmirGLO) obtained from RiboBio. PC3 cells were transfected with pmirGLO-FRMD6-WT or the mutant construct, and miR-mimic-NC or *miR-93-5p*-mimic by using Lipofectamine 3000 (Invitrogen). 48 h after transfection, Dual Glo Luciferase Reporter Gene Assay Kit (Yeasen, China) was used to examine the luciferase activity.

### Statistical analysis

SPSS 20 (IBM, USA) and GraphPad Prism 8 (GraphPad, USA) were used to perform all statistical analyses. The data were presented as means ± standard deviation (SD). Differences between groups were evaluated by carrying out a Student's *t* test or one-way ANOVA test. The Kaplan-Meier survival analysis was used for estimating the OS and PFS of patients. A *p* value < 0.05 was considered as statistical significance (*p < 0.05, **p < 0.01, ***p < 0.001). The schematic diagram was drawn using Figdraw (www.figdraw.com).

## Results

### Expression and prognostic relevance of m6A regulators in prostate cancer

In order to explore the expression pattern of m6A regulators and screen for the members with research value, an analysis of 18 m6A-related genes was carried out in PRAD cohort from TCGA. Significant difference of expression were found for 12 genes, including *RMB15*, *YTHDF1*, *YTHDF2*, *IGF2BP1*, *IGF2BP2*, *IGF2BP3*, *WTAP*, *HNRNPC*, *METTL3*, *HNRNPA2B1*, *FTO*, *METTL14*, *ZC3H13*, *YTHDC1*, *YTHDC2*, *YTHDF3*, *ALKBH5*, and *METT10D*, while the others showed no significant difference (Figure S1A, B). To further understand the prognostic relevance of the m6A regulators in PCa patients, we performed univariate Cox regression to analyze the expression level associated with PFS and OS (Figure S1C). The result presented a significant correlation between high level of *HNRNPA2B1* and poor PFS, as well as high expression of *YTHDC1* and poor OS.

### Expression of *HNRNPA2B1* is elevated in PCa and positively correlated with poor prognosis of prostate cancer patients

**Figure 1:**
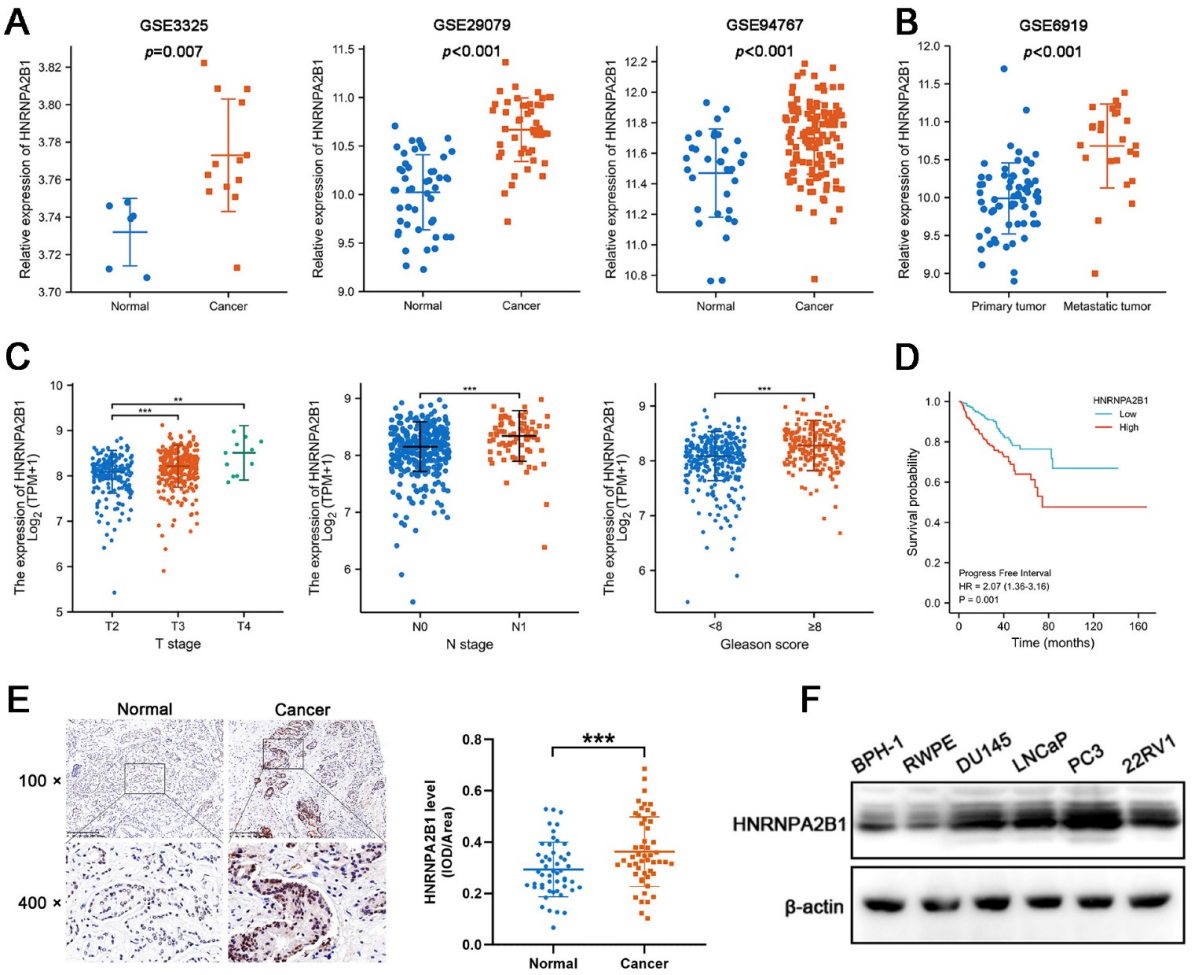
Expression of HNRNPA2B1 is elevated in PCa and positively correlated with poor prognosis of prostate cancer patients. (A) mRNA expression of HNRNPA2B1 in GEO datasets (GSE3325, GSE29079 and GSE94767) containing human prostate cancer samples and normal prostate tissues. (B) The expression level of HNRNPA2B1 in primary tumors versus metastatic samples (GSE6919). (C) The differential analysis of HNRNPA2B1 between different T (primary tumor) stage samples, different N (lymph node) stage samples and different Gleason score (<8 versus ≥8) samples in TCGA cohort. (D) Kaplan-Meier survival curve of HNRNPA2B1-high versus HNRNPA2B1-low for progression-free survival of patients with prostate cancer in TCGA cohort. (E) Representative HNRNPA2B1 IHC staining results of paired PCa and adjacent normal prostate tissues (scale bar, 200 μm) (left). Quantitative analysis of IHC staining according to the average integrated optical density per area (IOD/Area) (right). (F) Protein level of HNRNPA2B1 in BPH-1, RWPE-1, DU145, LNCaP, PC3 and 22RV1 analyzed by Western blotting with β-actin as the internal reference. Bar graphs are represented as mean ± SD; *P <0.05, **P<0.01, ***P<0.001.

Considering that the expression of *HNRNPA2B1* was significantly elevated in PCa and that high levels of *HNRNPA2B1* were correlated with a poor prognosis, we were motivated to investigate if *HNRNPA2B1* was involved in PCa development. We analyzed *HNRNPA2B1* expression in the GEO datasets (GSE3325, GSE29079, GSE94767, and GSE6919) of prostate cancer and normal prostate samples. In line with TCGA data, cancer tissues also expressed more HNRNPA2B1 than normal tissues (Figure 1A). Furthermore, metastatic PCa tumors also presented with higher *HNRNPA2B1* expression (Figure 1B). The correlation between *HNRNPA2B1* and clinicopathological characteristics, including cancer stage, lymphatic metastasis, and Gleason score, was then investigated in the prostate tissue data from TCGA. *HNRNPA2B1* was dramatically upregulated in patients with advanced stages, lymphatic metastasis, and high Gleason scores (Figure 1C). The Kaplan-Meier plot revealed that patients with high level of HNRNPA2B1 had poor PFS (Figure 1D). Moreover, we also observed an increase in HNRNPA2B1 levels in PCa tissues by performing immunohistochemistry analysis of 51 pairs of prostate tissues (Figure 1E). The association between the protein level of HNRNPA2B1 in PCa tissues and clinicopathological data was analyzed. A remarkable positive association was observed between HNRNPA2B1 expression and tumor stage as well as the grade of PCa (Table S6), while HNRNPA2B1 levels showed no significant association with the age of patients. We next detected upregulated protein levels of HNRNPA2B1 in PCa cells in comparison to normal prostatic epithelial cells (RWPE) and BPH cells (BPH-1) (Figure 1F). Thus, based on the above evidence, we concluded that *HNRNPA2B1* is frequently upregulated in PCa and implicated in a poor prognosis of patients with PCa.

### Knockout of *HNRNPA2B1* inhibits proliferation, migration, and invasion of PCa cells

**Figure 2:**
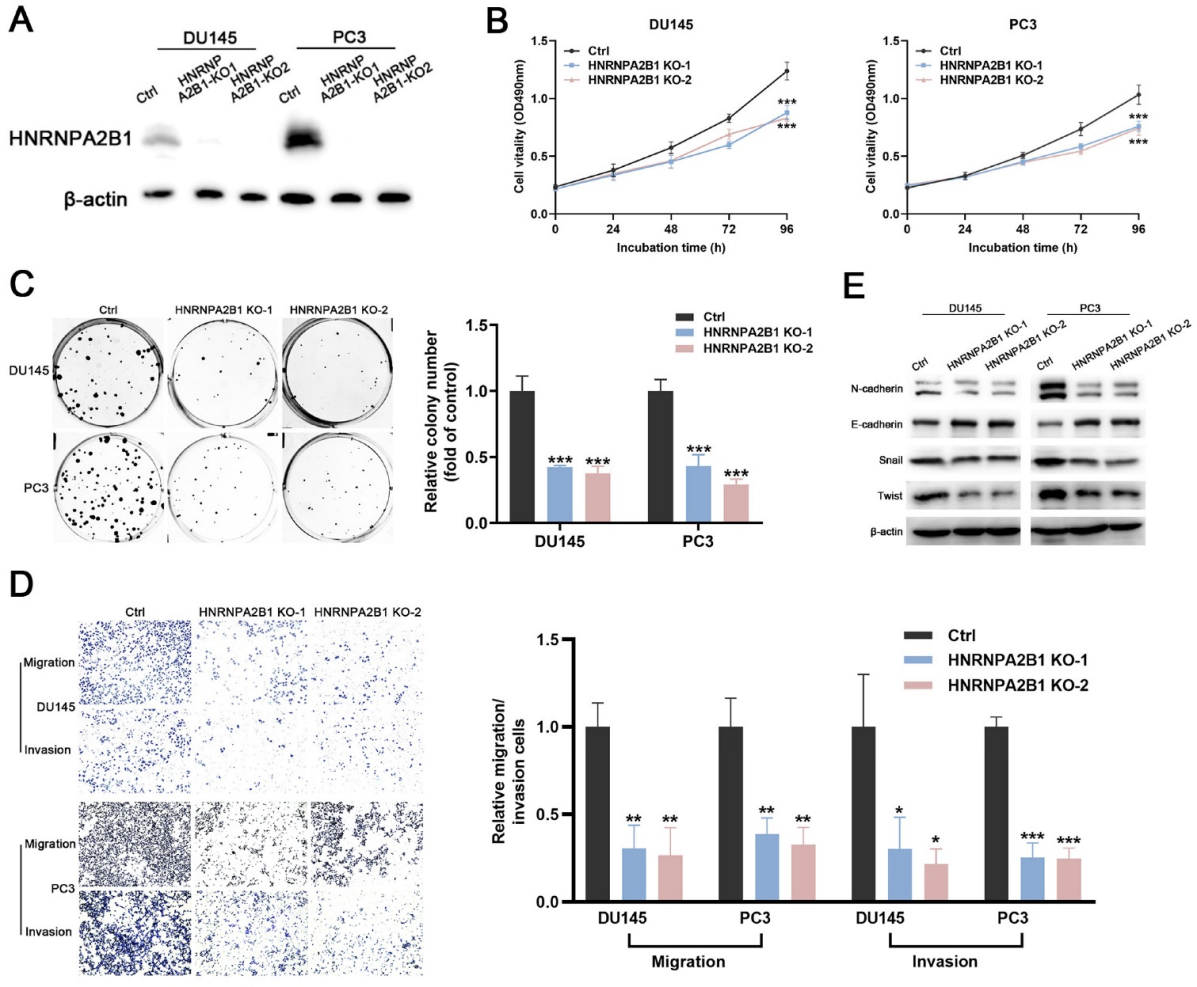
Knockout of HNRNPA2B1 inhibits prostate cancer cell proliferation, migration and invasion *in vitro*. (A) Western blot was performed to confirm HNRNPA2B1 knockout with CRISPR/Cas 9 in DU145 and PC3. (B) The growth curves of control and HNRNPA2B1 knockout DU145 (left) and PC3 (right) cell lines were analyzed using CCK8 assay. (C) Representative wells of colony formation assays of control and HNRNPA2B1 knockout DU145 and PC3 cells (left). Quantification of colony formation assay (right). (D) Trans-well migration and matrigel invasion assays of control and HNRNPA2B1 knockout DU145 and PC3 cells (representative wells were presented) (left). Quantification of migration and invasion cells in each field (right). (E) Analysis of EMT-related proteins in control and HNRNPA2B1 knockout cells by Western blot. Each experiment was conducted in triplicate; bar graphs are represented as mean ± SD; *P <0.05, **P<0.01, ***P<0.001.

The significant upregulation of *HNRNPA2B1* in prostate cancer and strong correlation between *HNRNPA2B1* and poor clinicopathological characteristics prompted us to further explore its role in tumor proliferation and invasion of PCa. DU145 and PC3 cell lines were selected for subsequent experiments, because of their high expression of HNRNPA2B1. A CRISPR/Cas9 system was used to knockout *HNRNPA2B1* in DU145 and PC3, and the depletion of HNRNPA2B1 was confirmed by western blot (Figure 2A). CCK-8 assays and colony formation assays demonstrated that HNRNPA2B1 depletion substantially impaired the proliferation and colony formation ability of DU145 and PC3 cells (Figure 2B, C). To examine the role of HNRNPA2B1 in migratory and invasive capacities of PCa cells, we performed transwell chamber assays with or without matrigel, respectively. *HNRNPA2B1* knockout significantly impaired migration and invasion of DU145 and PC3 cells (Figure 2D). Moreover, the epithelial-mesenchymal transition (EMT) is thought to be highly correlated with cell migratory and invasive capabilities, and therefore we detected the expression of EMT-associated proteins. There was an increase in the expression of E-cadherin in HNRNPA2B1 knockout cell lines, whereas N-cadherin, Twist, and Snail were downregulated (Figure 2E).

### Knockout of *HNRNPA2B1* inhibits tumor growth and metastasis *in vivo*

**Figure 3:**
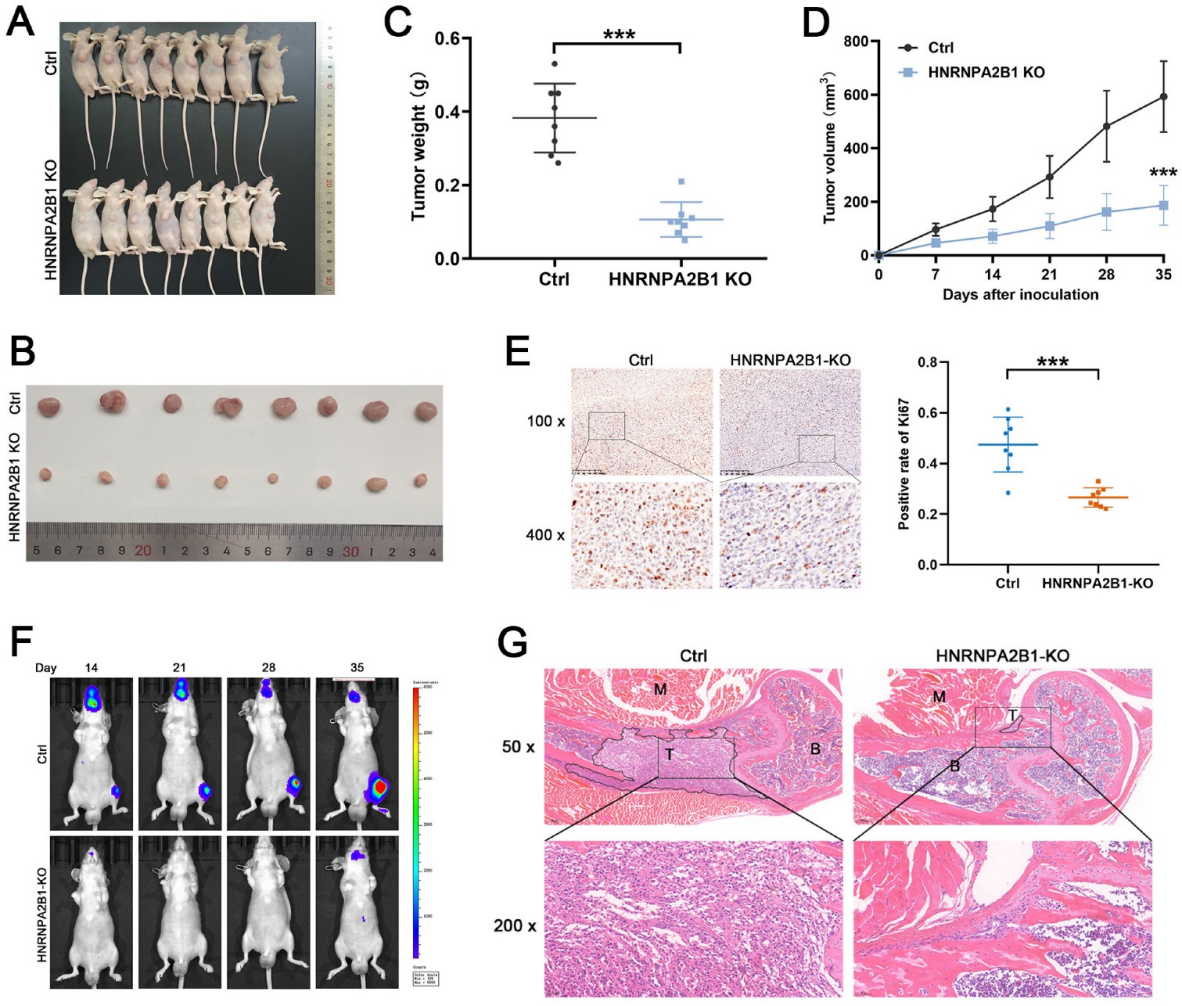
Knockout of HNRNPA2B1 inhibits prostate cancer growth and metastasis *in vivo*. (A) Images of subcutaneous tumor models at 5 weeks after injection of PC3-ctrl and PC3-HNRNPA2B1-knockout cells. (n=8) (B) Images of mice-bearing xenografts. (C) Tumor weights of two different groups. (D) Tumor growth curve of subcutaneous tumor volume data. (E) Representative IHC images of Ki67 staining of subcutaneous tumor sections (100 x and 400 x) (left). Quantitation of Ki67-positivity (right). (F) Representative images of tumor bioluminescence of mice following intracardiac injection with PC3-ctrl and PC3-HNRNPA2B1-knockout cells at 14, 21, 28 and 35 days (n=5). (G) Representative HE staining images of bone metastasis derived from proximal tibia corresponding to the bioluminescence images. Skeletal muscle (M), tumor (T), bone marrow (B) (50x and 200x). Bar graphs are represented as mean ± SD; *P <0.05, **P<0.01, ***P<0.001.

To explore the functional role of *HNRNPA2B1* in PCa *in vivo*, *HNRNPA2B1* knockout PC3 cells and control PC3 cells were used to construct a xenograft tumor model. During the observation of the xenograft growth, we noted that mice injected with *HNRNPA2B1* knockout cells developed significantly smaller tumors than those in the control group (Figure 3A, B).

The tumor growth curves and tumor weight data were also analyzed. Consistent with the *in vitro* CCK-8 and colony formation assays, the results showed statistically significant differences demonstrating that knockout of *HNRNPA2B1* inhibited xenograft tumor growth (Figure 3C, D). Furthermore, to validate the level of Ki-67 in xenograft tumors, the xenograft tissues were paraffin-embedded and underwent immunohistochemical staining for Ki-67. The result suggested that the Ki-67 levels were significantly decreased in the xenograft of the experimental group (Figure 3E).

The intracardiac injection metastatic models were constructed using luciferase-expressing PC3 (*HNRNPA2B1* knockout and control) cells to evaluate the effects of *HNRNPA2B1* knockout on bone metastasis. The metastasis condition was monitored using a bioluminescence imaging system. Compared with the HNRNPA2B1 knockout group, the number and volume of metastatic tumors in the control group were significantly higher (Figure 3F). The bone metastatic tissues were isolated and stained using hematoxylin and eosin staining to confirm and locate the formation of metastasis (Figure 3G). To summarize, knocking out *HNRNPA2B1* significantly inhibited tumor growth and bone metastasis *in vivo*.

### HNRNPA2B1 promotes *miR-93-5p* processing in an m6A-dependent manner

**Figure 4:**
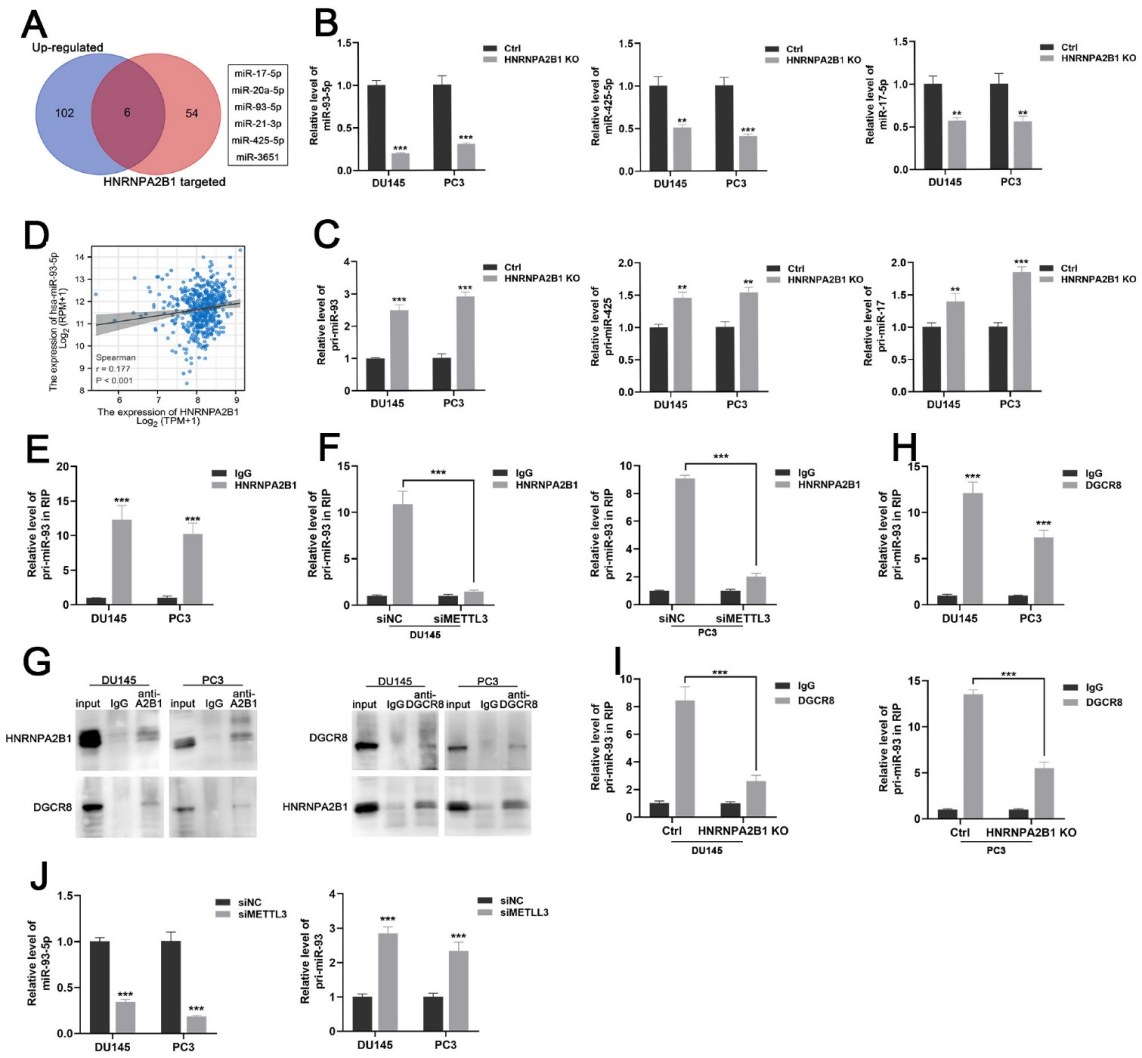
HNRNPA2B1 promotes miR-93-5p processing in a m6a-dependent manner. (A) Venn diagram presenting the overlap between upregulated miRNAs in PCa dataset of TCGA cohort and HNRNPA2B1-modulated miRNAs. (B) qPCR analyses show the relative expression of mature miR-93-5p, miR-425-5p and miR-17-5p in parental and HNRNPA2B1 knockout PCa cell lines. (C) qPCR analyses show the relative expression of pri-miR-93, pri-miR-425 and pri-miR-17 in parental and HNRNPA2B1 knockout PCa cell lines. (D) The scatterplot image shows the correlation between HNRNPA2B1 and miR-93-5p in TCGA cohort. (E) Result of RIP-qPCR assay detecting pri-miR-93 enriched by antibody of HNRNPA2B1 and IgG in DU145 and PC3 cells. (F) RIP-qPCR assay detecting pri-miR-93 enriched by antibody of HNRNPA2B1 and IgG in PCa cells transfected with METTL3 siRNA. (G) Lysates from DU145 and PC3 cells were subjected to Co-IP with anti-HNRNPA2B1 and anti-DGCR8 (with IgG as negative control). The blots were probed with anti-HNRNPA2B1 and anti-DGCR8 respectively. (H) RIP-qPCR assays detecting pri-miR-93 enriched by antibody of DGCR8 and IgG in DU145 and PC3 cells. (I) RIP-qPCR assay detecting pri-miR-93 enriched by antibody of DGCR8 and IgG in HNRNPA2B1-knockout PCa cells. (J) qPCR analyses show the changes of mature miR-93-5p and pri-miR-93 in DU145 and PC3 transfected with METTL3 siMETTL3. Each experiment was conducted in triplicate; bar graphs are represented as mean ± SD; *P <0.05, **P<0.01, ***P<0.001.

HNRNPA2B1 has been reported as a nuclear m6A reader mediating pri-miRNA processing and miRNA maturation events by interacting with the microRNA microprocessor complex protein DGCR8 12, 19. To investigate which set of miRNAs were regulated by HNRNPA2B1 in prostate cancer, we combined the HNRNPA2B1-modulated miRNA data from a previous study 12 and TCGA data of upregulated miRNAs in prostate cancer, and obtained six potential downstream miRNAs that intersected on the Venn diagram (Figure 4A). In an attempt to identify the prognostic relevance of these six miRNAs in PCa patients, we analysed the miRNA expression profiles of TCGA-PRAD cohort with available PFS and OS data. The result presented a significant correlation between high level of *miR-93-5p* and poor PFS, while no correlation was identified between other miRNAs expression level and prognosis (Figure S2A-B). We next performed qRT-PCR analysis to quantify the levels of these six mature miRNAs and found that only *miR-17-5p*, *miR-93-5p*, and *miR-425-5p* were decreased in *HNRNPA2B1* knockout prostate cancer cells (Figures 4B and S2C). As an indication of the block in miRNA maturation, levels of their primary transcripts were correspondingly increased (Figure 4C). Considering the most prominent alterations were observed in mature *miR-93-5p* and *pri-miR-93*, *miR-93-5p* might be the HNRNPA2B1-modulated miRNA in PCa. We also found a significant positive correlation between *miR-93-5p* and HNRNPA2B1 through analyzing TCGA-PRAD dataset (Figure 4D). Next, we found that by using antibodies to HNRNPA2B1 in PCa cell lysates, *pri-miR-93* was enriched (Figure 4E), while depleting METTL3 inhibited this interaction (Figures 4F and S2D, E), suggesting that the m6A placement on *pri-miR-93* by the m6A reader METTL3 was necessary for the recognition of HNRNPA2B1. RNA-binding protein DGCR8 was shown to be a component of the microprocessor complex, participating in the processing of pri-miRNAs 20. Co-immunoprecipitation identified the interaction between HNRNPA2B1 and DGCR8 in prostate cancer cells (Figure 4G). *Pri-miR-93* was also enriched using antibodies to DGCR8 (Figure 4H), while *HNRNPA2B1* knockout dramatically inhibited the interaction between DGCR8 and *pri-miR-93* (Figure 4I). Furthermore, *METTL3* knockdown also reduced the level of *miR-93-5p* and induced the accumulation of *pri-miR-93* (Figure 4J). These data collectively indicated that HNRNPA2B1 facilitated the processing of *pri-miR-93* by recruiting DGCR8 in an m6A-dependent manner.

### HNRNPA2B1/*miR-93-5p* exert oncogenic roles in PCa cells

**Figure 5:**
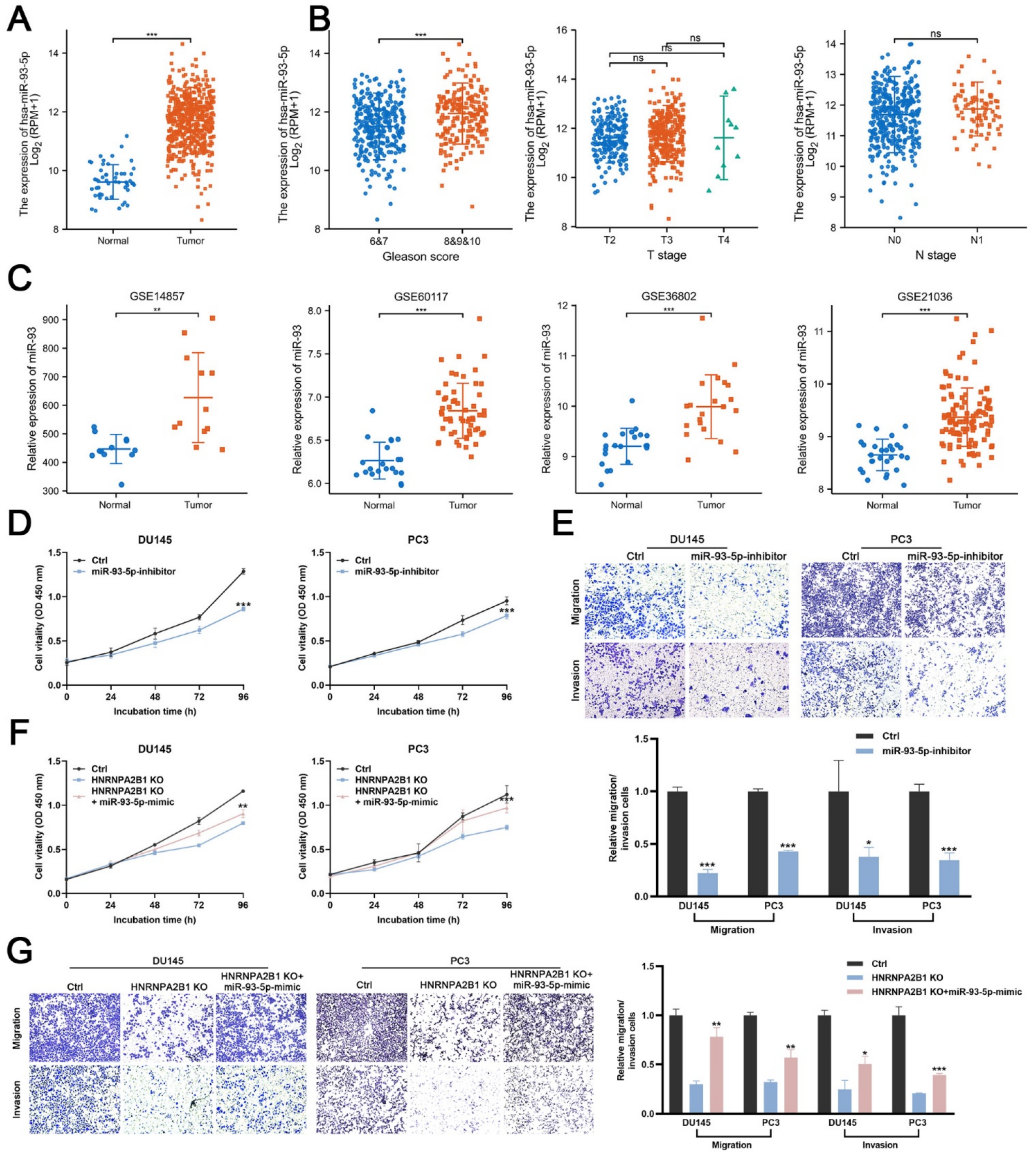
HNRNPA2B1/miR-93-5p exert oncogenic roles in PCa cells (A) The differential analysis of miR-93-5p level between human prostate cancer samples and normal prostate tissues in TCGA cohort. (B) The differential analysis of miR-93-5p level between different T (primary tumor) stage samples, different N (lymph node) stage samples and different Gleason score (<8 versus ≥8) samples in TCGA cohort. (C) Differential analysis of miR-93-5p in GEO datasets containing miRNA microarray data of human prostate cancer samples and normal prostate tissues. (D) Growth curves show the proliferation ability of DU145 (left) and PC3 (right) cells transfected with miR-93-5p inhibitor. (E) Trans-well migration and matrigel invasion assays of DU145 and PC3 cells with transfection of miR-93-5p inhibitor (representative wells were presented) (up). Quantification of migration and invasion cells in each field (low). (F) Growth curves show the proliferation ability of HNRNPA2B1-knockout DU145 (left) and PC3 (right) cells transfected with miR-93-5p mimic. (G) Trans-well migration and matrigel invasion assays of HNRNPA2B1-knockout DU145 and PC3 cells transfected with miR-93-5p mimic (representative wells were presented) (left). Quantification of migration and invasion cells in each field (right). Each experiment was conducted in triplicate; bar graphs are represented as mean ± SD; *P <0.05, **P<0.01, ***P<0.001.

Next, we evaluated the *miR-93-5p* level in PCa datasets (TCGA, GSE14857, GSE21036, GSE36802, and GSE60117). The expression levels of *miR-93-5p* were significantly increased in prostate cancer samples (Figure 5A, C). The correlation between the level of *miR-93-5p* and clinicopathological features of PCa from TCGA datasets were also been analyzed. Upregulated *miR-93-5p* was observed in samples from patients with high Gleason scores (Figures 5B). To verify whether *miR-93-5p* functioned as a downstream factor of HNRNPA2B1 and to recapitulate the effects of *HNRNPA2B1* silencing, we transfected DU145 and PC3 cells with a *miR-93-5p* inhibitor. Consistent with knockout of *HNRNPA2B1*, the cell proliferation and colony formation ability of PCa cells treated with the *miR-93-5p* inhibitor were significantly decreased in comparison to the control group (Figures 5D and S2F). In terms of cell migration and invasion, a significant reduction of the cell penetration rate of DU145 and PC3 was observed upon inhibition of *miR-93-5p* (Figure 5E). Consistently, western blot analysis demonstrated that the EMT was markedly changed by *miR-93-5p* inhibition (Figure S2G). Next, we examined whether *miR-93-5p* mimic could rescue the effects of *HNRNPA2B1* knockout. Of note, *miR-93-5p* mimic partially rescued the impaired cell proliferation ability and motility induced by *HNRNPA2B1* depletion (Figures 5F, G and S2H). Collectively, the above data indicated that the oncogenic effects of HNRNPA2B1 in PCa are, at least partially, modulated via promoting *miR-93-5p* processing.

### HNRNPA2B1/*miR-93-5p* directly target FRMD6 in prostate cancer

**Figure 6:**
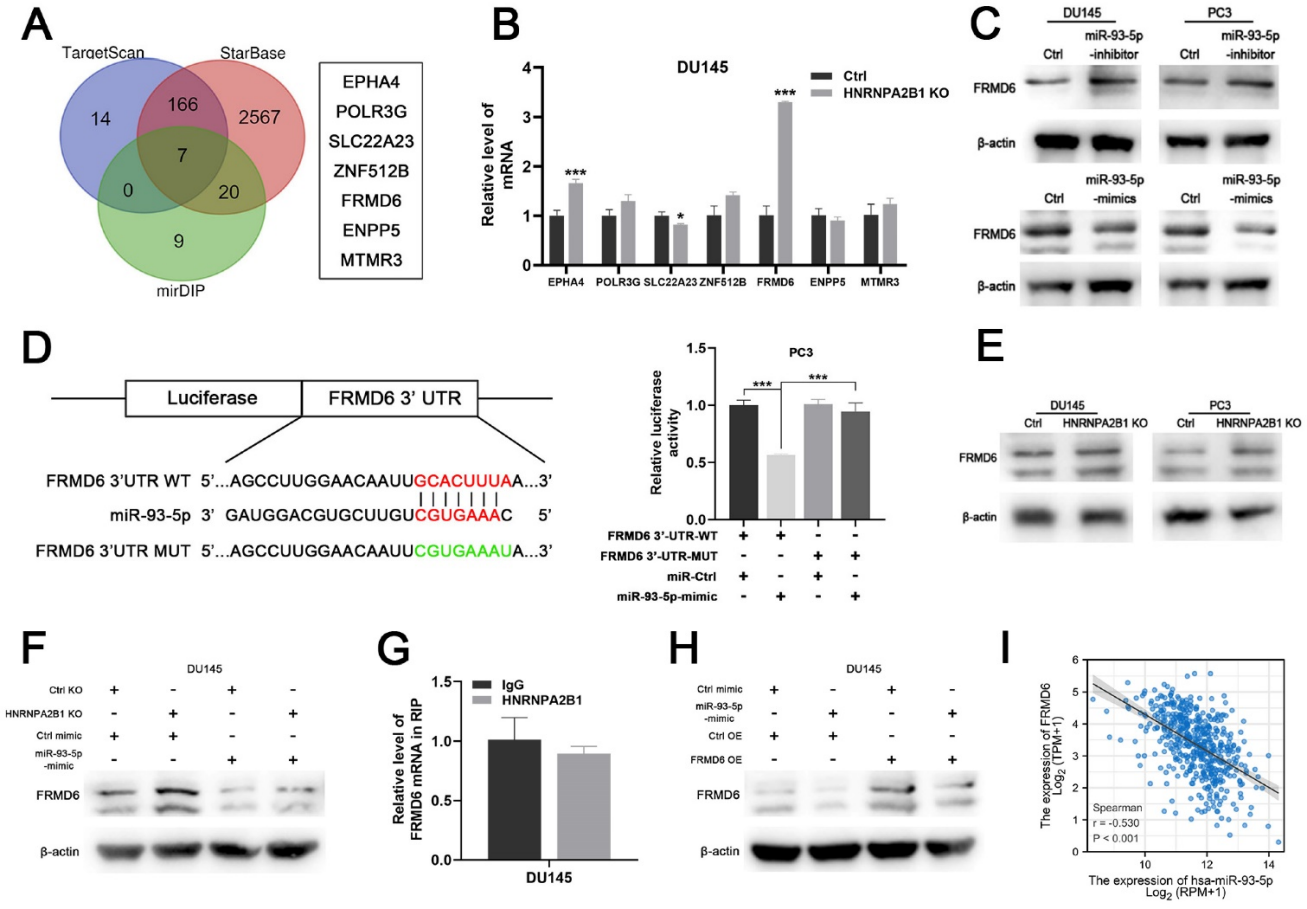
HNRNPA2B1/miR-93-5p targets FRMD6 directly in prostate cancer (A) Venn diagram presenting the overlap of 3 common miRNA target prediction databases (left). 7 potential miR-93-5p target genes (right). (B) qPCR analyses show mRNA level changes of EPHA4, POLR3G, SLC22A23, ZNF512B, FRMD6, ENPP5 and MTMR3 in HNRNPA2B1-knockout DU145 cell. (C) Western blot analysis of FRMD6 protein level changes in DU145 and PC3 cells transfected with miR-93-5p inhibitor or mimic. (D) Dual luciferase reporter gene assay in PC3 cell co-transfected with the dual luciferase reporter containing FRMD6 wild-type 3' UTR or mutant and the mimic of miR-93-5p or mimic-ctrl. (E) Western blot analysis of FRMD6 protein levels in control and HNRNPA2B1-knockout DU145 and PC3 cells. (F) Western blot analysis of FRMD6 protein changes in HNRNPA2B1-knockout DU145 cell transfected with miR-93-5p mimic. (G) RIP-qPCR assay detecting mRNA of FRMD6 enriched by antibody of HNRNPA2B1 and IgG in DU145. (H) Western blot analysis of FRMD6 protein changes in FRMD6-overexpressing DU145 cell transfected with miR-93-5p mimic. (I) The scatterplot image showing the correlation between FRMD6 and miR-93-5p in TCGA cohort. Each experiment was conducted in triplicate; bar graphs are represented as mean ± SD; *P <0.05, **P<0.01, ***P<0.001.

To identify the downstream mechanism of HNRNPA2B1/*miR-93-5p*, TargetScan 21, STARbase 22, 23, and mirDIP 24 were used to predict the potential targets of *miR-93-5p*. After taking the intersection of the results of the three algorithms, a total of seven genes, including *EPHA4*, *POLR3G*, *SLC22A23*, *ANF512B*, *FRMD6*, *ENPP5*, and *MTMR3*, were predicted to be *miR-93-5p* target genes (Figure 6A). We next performed differential expression analysis and survival analysis of the 7 candidates in TCGA-PRAD cohort. The results demonstrated that EPHA4 and FRMD6 were significantly downregulated in PCa (Figure S3A) and lower level of FRMD6 was associated with poor PFS of PCa patients (Figure S3B). Among these candidate genes, FERM domain-containing protein 6 (*FRMD6*) further intrigued us because of its striking expression fold change upon *HNRNPA2B1* knockout (Figure 6B). Western blot analysis showed that *miR-93-5p*-mimic and *miR-93-5p*-inhibitor seperately decreased and increased FRMD6 protein expression levels, respectively (Figure 6C). Additionally, we performed a dual luciferase assay to determine whether miR-93-5p directly interacts with the 3'UTR of FRMD6 mRNA. The results demonstrated that the *miR-93-5p* mimic reduced the luciferase activity, confirming the direct interaction between *miR-93-5p* and FRMD6 (Figure 6D). Considering that *miR-93-5p* is positively regulated by HNRNPA2B1, we hypothesized that depletion of HNRNPA2B1 would recapitulate the inhibitory effects of *miR-93-5p* on FRMD6. As expected, *HNRNPA2B1* knockout significantly increased *FRMD6* expression (Figure 6E), and the additional transfection with *miR-93-5p*-mimic rescued the upregulation of *FRMD6* (Figure 6F). Because a previous study reported that HNRNPA2B1 directly bound to mRNA transcripts and enhanced their stability, we performed an RIP assay to detect if HNRNPA2B1 interacted with the mRNA of *FRMD6*. The results showed no significant difference in *FRMD6* transcripts precipitated by IgG and HNRNPA2B1 antibody (Figure 6G). We constructed *FRMD6*-overexpressing DU145 and PC3 cell lines (Figure S3C). The downregulation of *FRMD6* induced by transfection of *miR-93-5p*-mimic was partially rescued by *FRMD6* overexpression (Figure 6H). Furthermore, we also found a significant negative correlation between the level of *miR-93-5p* and *FRMD6* through analyzing TCGA-PRAD database (Figure 6I). Collectively, these results confirmed that FRMD6 is the direct target of *miR-93-5p* and can be regulated by HNRNPA2B1 indirectly.

### *FRMD6* is a tumor suppressor in PCa

**Figure 7:**
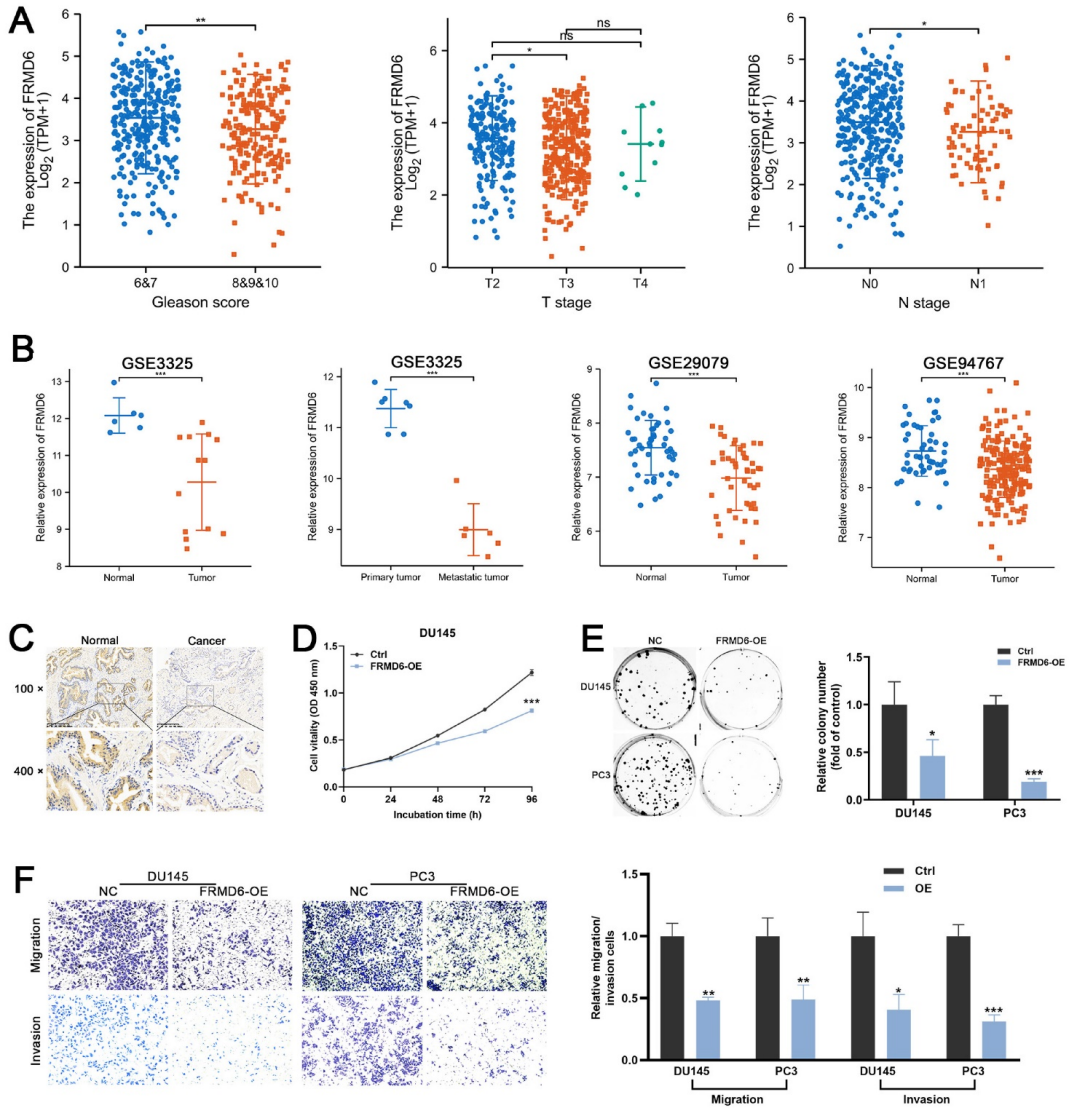
FRMD6 plays a role of tumor suppressor in PCa (A) The differential analysis of FRMD6 between different T (primary tumor) stage samples, different N (lymph node) stage samples and different Gleason score (<8 versus ≥8) in TCGA cohort. (B) Differential analysis of FRMD6 in GEO datasets (GSE3325, GSE29079 and GSE94767) containing human primary prostate cancer, metastatic prostate cancer samples and normal prostate tissues. (C) Representative FRMD6 IHC staining images of paired PCa and adjacent normal prostate tissues (scale bar, 200 μm). (D) Growth curves show the proliferation ability of FRMD6-overexpressing DU145 cells. (E) Representative wells of colony formation assays of parental and FRMD6-overexpressing DU145 and PC3 cells (left). Quantification of colony formation assay (right). (F) Trans-well migration and matrigel invasion assays of parental and FRMD6-overexpressing DU145 and PC3 cells (representative wells were presented) (left). Quantification of migration and invasion cells in each field (right). Each experiment was conducted in triplicate; bar graphs are represented as mean ± SD; *P <0.05, **P<0.01, ***P<0.001.

To detect the role of *FRMD6* in PCa, we analyzed its expression information in TCGA and GEO databases. TCGA analysis demonstrated that PCa tissues expressed lower levels of *FRMD6* in comparison to normal prostate tissues (Figure S3A) and PCa tissues with adverse pathological features (Gleason ≥ 8, T ≥ 3 and N1) expressed lower levels of *FRMD6* (Figure 7A). Analysis of GEO datasets (GSE29079, GSE94767, and GSE3325) also showed that *FRMD6* was significantly downregulated in prostate cancer tissues and metastatic tumor tissues (Figures 7B). Immunohistochemistry analysis of our paired prostate tissues also verified the downregulation of FRMD6 in prostate cancer (Figure 7C).

Moreover, we found lower level of FRMD6 in PCa cells in comparison to normal prostatic epithelial cells (RWPE) and BPH cells (BPH-1) (Figure S3D). To examine whether FRMD6 functioned as a tumor inhibiting factor in PCa cells, we constructed *FRMD6*-overexpressing DU145 and PC3 cell lines (Figure S3C). The CCK-8 assay results suggested that overexpression of *FRMD6* dramatically inhibited proliferation (Figures 7D and S3E). We also found that overexpression of *FRMD6* in PCa cells weakened the ability for colony formation (Figure 7E). The results of transwell assays also demonstrated that the forced expression of *FRMD6* clearly impaired the mobility of PCa cells (Figure 7F), which was consistent with lower levels of *FRMD6* in metastatic tumors (Figure 7B). Thus, FRMD6 plays a suppressor role in PCa, and works as a downstream factor of HNRNPA2B1/*miR-93-5p*.

## Discussion

Heterogeneous nuclear ribonucleoproteins (hnRNPs) represent a large group of RNA-binding proteins (RBPs) located in the nucleus of eukaryotic cells, and include approximately 30 members 25. Given that hnRNPs are mainly responsible for processing and stabilizing freshly transcribed primary RNAs, hnRNPs play an important role in nucleic acid metabolism 26, 27. As a member of the hnRNP family, HNRNPA2B1 is composed of A2 and B1 proteins, which differ from each other by only 12 amino acids. HNRNPA2B1 has been reported to be involved in almost every step of RNA metabolism by binding to a series of specific RNA sequences, thereby influencing the expression of a vast number of downstream genes, and regulating several cellular biological processes 13.

In a previous study, HNRNPA2B1 was identified as a nuclear m6A reader that functioned as an effector of the m6A marker added by METTL3 and an adaptor of the microprocessor complex participating in the processing of a set of pri-miRNAs 12, 19. HNRNPA2B1 was found to play an oncogenic role in malignant phenotypes of various malignancies. In multiple myeloma, HNRNPA2B1 was shown to recognize the m6A site of *ILF3* and enhanced the stability of its mRNA transcript to promote cell proliferation 28. HNRNPA2B1 also facilitated the stabilization of *TCF7L2* mRNA in an m6A-dependent manner to maintain cetuximab resistance and promote progression of colorectal cancer 29. The present study revealed that *HNRNPA2B1* was significantly elevated in prostate cancer and the high level of *HNRNPA2B1* was strongly associated with poor prognosis. Furthermore, metastatic PCa tissues presented higher level of HNRNPA2B1 compared to primary PCa tissues. *In vitro* cellular functional experiments showed that *HNRNPA2B1* knockout remarkably inhibited a series of phenotypes, including proliferation, invasion, and migration. In vivo subcutaneous xenograft tumor model revealed that *HNRNPA2B1* depletion significanly suppress tumorigenic ability of prostate cancer. Since prostate cancer most commonly metastasizes to bone 30, we selected intracardiac injection metastatic models to simulate the process of PCa cell dissemination* in vivo*. The knockout of HNRNPA2B1 also inhibited bone metastasis in nude mice. Therefore, these results identify the importance of HNRNPA2B1 in prostate cancer progression and metastasis.

It is of note that the mechanism behind HNRNPA2B1 promoting the progression and metastasis of PCa is not fully elucidated. Alaron et al. proposed that m6A functioned as an important post-transcriptional modification that promoted the maturation of miRNAs 12. Specifically, HNRNPAB21 acted as a reader of the m6A marker on pri-miRNAs that were methylated by METTL3, and interacted with the microRNA microprocessor complex protein DGCR8, promoting the maturation of a group of miRNAs 12. In our study, we identified the intersection of this set of miRNAs and the upregulated miRNAs in PCa from TCGA database and obtained six potential downstream miRNAs of HNRNPA2B1. Then, we validated the level of these miRNAs and their primary transcripts in *HNRNPA2B1*-knockout cell lines, and observed the most striking changes in *miR-93-5p* and *pri-miR-93*. We also found that *miR-93-5p* expression was significantly higher in PCa tissues compared to normal prostate tissues and in high grade PCa compared to low grade tumor tissues, and that the elevated level correlated with a poorer PFS of patients. Next, under our experimental conditions, we verified the interaction between HNRNPA2B1 and *pri-miR-93* by RIP assays, an interaction that was weakened by depletion of METTL3. These particular results indicated that HNRNPA2B1 accelerated the maturation of *miR-93-5p* in an m6A-dependent manner in prostate cancer.

Accumulating evidence has demostrated that a number of miRNAs are extensively involved in modulation of cancer cell prolifendicated that ration and invasion by inhibition of their downstream target genes 31, 32. Elevated levels of *miR-93-5p* have been reported in multiple cancers and were correlated to tumor development and progression. In gastric cancer, *miR-93-5p* played an oncogenic role by activating the STAT3 pathway, and upregulation of *miR-93-5p* was found to be associated with shorter OS of patients 33. Furthermore, Huang et al. found that knockdown of *miR-93-5p* in small cell lung cancer cells facilitated chemosensitivity 34. Another bioinformatics analysis study proposed that *miR-93-5p* might play a role of oncogene in PCa 35, but the authors did not investigate the upstream regulators or downstream target genes of *miR-93-5p in PCa*. In the present research, our findings further confirmed that, in terms of malignant phenotypes, *miR-93-5p* interference successfully replicated the inhibiting effect of *HNRNPA2B1* knockout, and transfection of *miR-93-5p* mimics partially rescued the effect of *HNRNPA2B1* silencing in prostate cancer cell lines. To identify the major target genes of HNRNPA2B1/*miR-93-5p* in prostate cancer, we performed an integrative analysis of StarBase, TargetScan, and miRDIP database and obtained seven candidate target genes. After further validation through qRT-PCR and dual luciferase reporter assays, we confirmed that *FRMD6* was the direct target of *miR-93-5p* in prostate cancer and was selected for subsequent research. Shen et al. also reported that the 3'UTR of *FRMD6* contained complementary binding sites of *miR-93-5p* using bioinformatics prediction in cervical cancer, which was consistent with our findings 36.

FRMD6 was previously validated as a key upstream regulator of the Hippo signaling pathway that exerted an anti-tumor effect in several human cancers 37. A recent study demostrated that FRMD6 had a tumor suppressor role by suppressing the activation of carcinogenic YAP1 in thyroid cancer 38. While Haldrup et al. reported that *FRMD6* worked as a tumor suppressor gene in prostate cancer 39, they did not explore the upstream regulatory mechanisms. Here, we found that *HNRNPA2B1* and *FRMD6* were negatively correlated in prostate cancer. Transfection of *miR-93-5p*-mimics partially restored the upregulation of *FRMD6* induced by *HNRNPAB21* knockout. Bioinformatics analysis demonstrated that PCa tissues expressed lower levels of *FRMD6* in comparison to normal prostate tissues and metastatic PCa tissues expressed even less *FRMD6* compared to primary PCa samples. Cellular functional experiments were performed using *FRMD6*-overexpressing prostate cancer cell lines. Consistent with previous findings, FRMD6 exhibited an inhibitory effect on cell proliferation and invasion.

**Figure 8:**
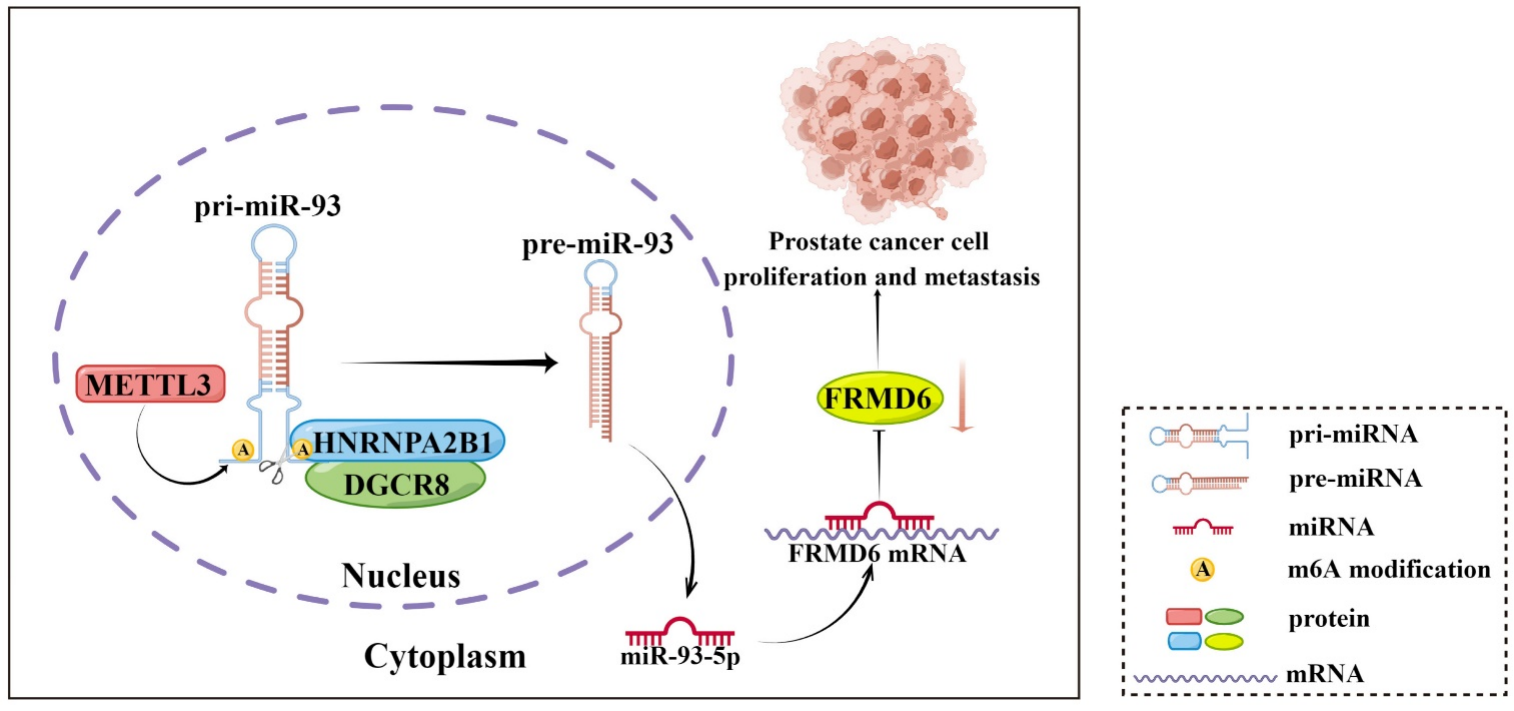
Schematic diagram (by Figdraw) of our findings in this study.

In conclusion, our present study showed that *HNRNPA2B1* expression was highly increased in prostate cancer and associated with poor prognosis of prostate cancer patients. Moreover, results from a series of functional assays demonstrated that HNRNPA2B1 promoted tumor growth and metastasis. As for the mechanisms of these functions, we found that HNRNPA2B1 downregulated *FRMD6*, a tumor suppressor, by accelerating the maturation of *miR-93-5p* in an m6A-dependent manner. These observations presented an axis of HNRNPA2B1/*miR-93-5p*/FRMD6 underlying PCa development and metastasis (Figure 8), and provided potential prognostic biomarkers or therapeutic targets in PCa.

## Supplementary Material

Supplementary figures and tables.

## Abbreviations

HNRNPA2B1: Heterogeneous nuclear ribonucleoprotein A2/B1
PCa: Prostate cancner
pri-miRNA: Primary microRNA
METTL3: Methyltransferase Like Protein 3
DGCR8: DiGeorge syndrome critical region gene 8
FRMD6: Methyltransferase Like Protein 3
CRIPSR: Clustered Regularly Interspaced Short Palindromic Repeats
m6A: N6-methyladenosine
TCGA: The Cancer Genome Atlas
GEO: Gene Expression Omnibus
IHC: Immunohistochemistry
KO: Knockout
RIP: RNA immunoprecipitation
Co-IP: Co-immunoprecipitation
CCK8: Cell counting kit-8
qRT-PCR: Quantitative real-time PCR
