# Supplementary material for: Activation of the HNRNPA2B1/miR-93-5p/FRMD6 axis facilitates prostate cancer progression in an m6A-dependent manner
Source: J Cancer. 2023 May 8;14(7):1242–56. doi: 10.7150/jca.83863 (PMC10197942; doi:10.7150/jca.83863)
Supplement: Supplementary file 1 — Supplementary figures and tables. [file jcav14p1242s1.pdf]

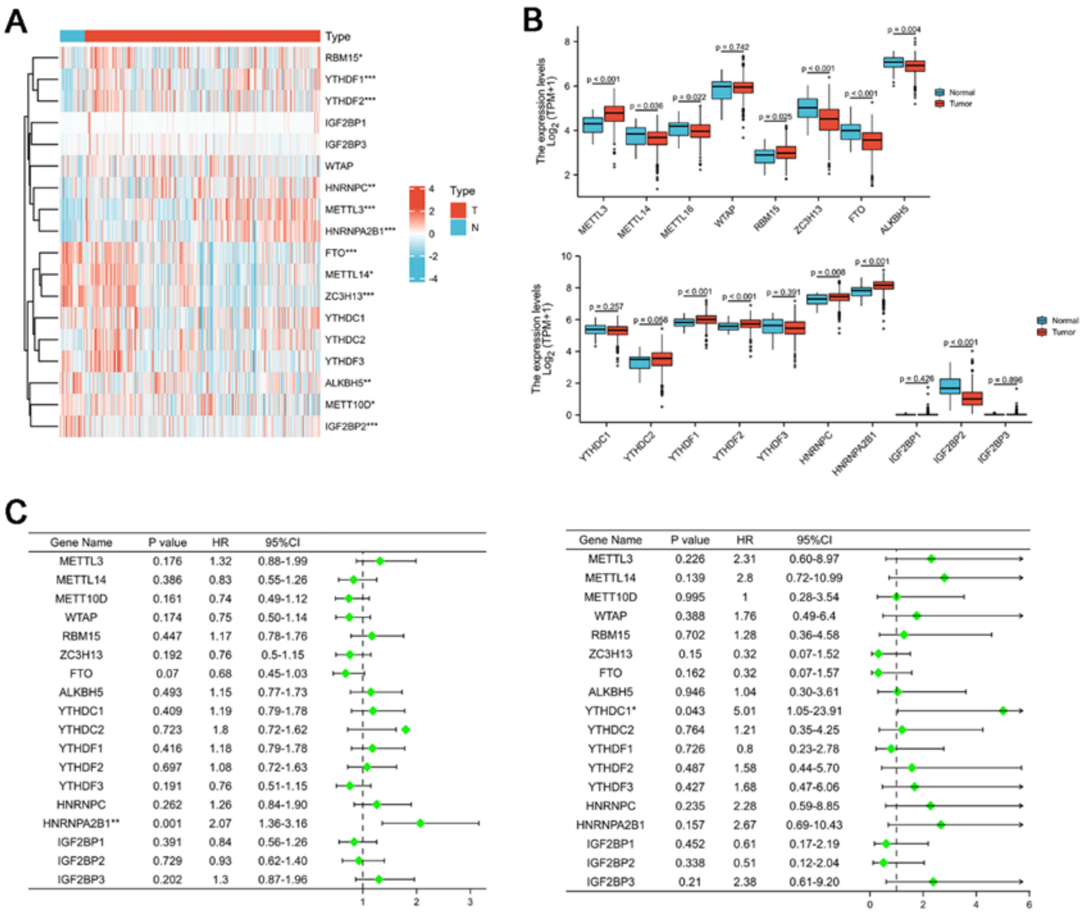

Figure S1. The expression and prognosis of 18 m6A regulators in PCa from TCGA database.

(A, B) Heatmap and bar charts visualized upregulated and downregulated m6A regulators in PCa specimens (T) and normal prostate specimens (N). In the heatmap, red means upregulated specimens and blue means downregulated specimens. (C) Univariate Cox regression analysis of the progression-free survival (PFS) time and overall survival (OS) time of 18 m6a regulators; *P <0.05, **P<0.01, ***P<0.001.

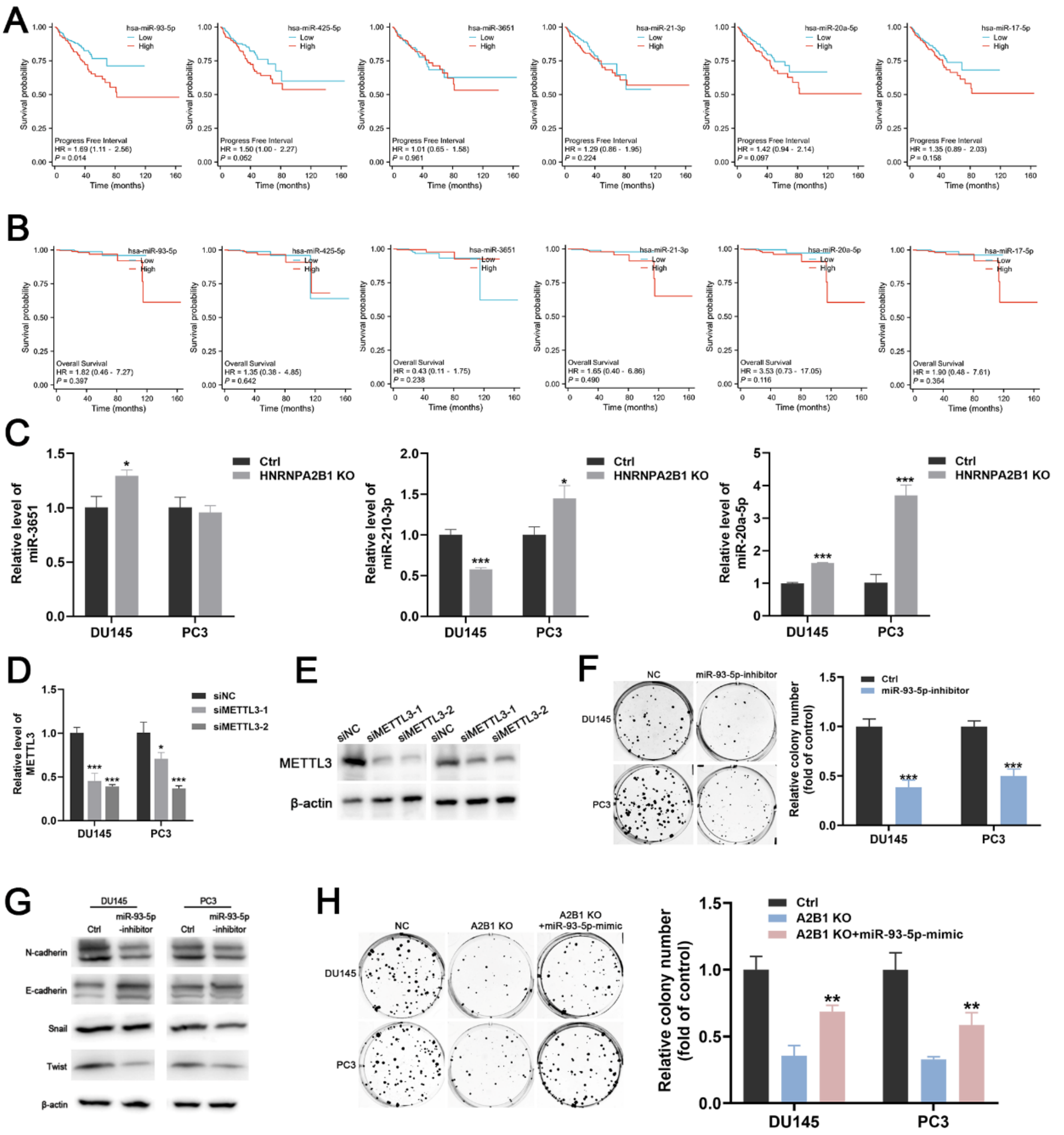

Figure S2. Supplementary figure 2.

(A) Kaplan-Meier survival analysis for progression-free survival of patients with prostate cancer in TCGA cohort based on differential expression of miR-93-5p, miR-425-5p, miR-3651, miR-21-3p, miR-20a-5p and miR-17-5p. (B) Kaplan-Meier survival analysis for overall survival of patients with prostate cancer in TCGA cohort based on differential expression of miR-93-5p, miR-425-5p, miR-3651, miR-21-3p, miR-20a-5p and miR-17-5p. (C) qPCR assays showing the changes of mature miR-3651, miR-210-3p and miR-20a-5p. (D) qPCR assays confirming METTL3 knockdown by siRNAs in DU145 and PC3. (E) Western blot confirming METTL3 knockdown by siRNAs in DU145 and PC3. (F) Representative wells of colony formation assays of DU145 and

PC3 cells transfected with miR-93-5p inhibitor (up). Quantification of colony formation assay (low). (G) Analysis of EMT-related proteins in PCa cells with transfection of miR-93-5p inhibitor by Western blot. (H) Representative wells of colony formation assays of HNRNPA2B1-knockout DU145 and PC3 cells transfected with miR-93-5p mimic (left). Quantification of colony formation assay (low) (right). Each experiment was conducted in triplicate; bar graphs are represented as mean ± SD; *P <0.05, **P<0.01, ***P<0.001.

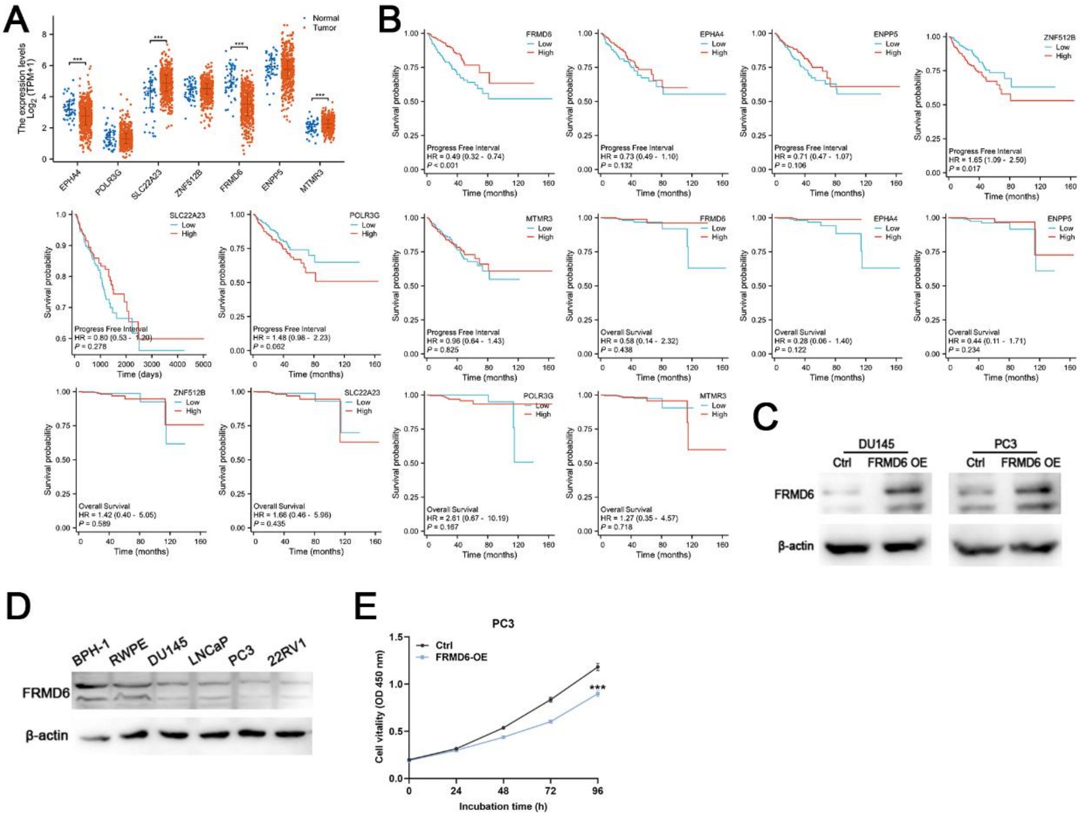

Figure S3. Supplementary figure 3.

(A) The differential expression analysis of EPHA4, POLR3G, SLC22A23, ZNF512B, FRMD6, ENPP5 and MEMR3 between normal prostate tissues and prostate cancer samples in TCGA cohort. (B) Kaplan-Meier survival analysis for overall survival and progression-free survival of patients with prostate cancer in TCGA cohort based on differential expression of EPHA4, POLR3G, SLC22A23, ZNF512B, FRMD6, ENPP5 and MEMR3. (C) Western blot confirming FRMD6 overexpression in FRMD6-overexpressing DU145 and PC3 cells. (D) Protein level of FRMD6 in BPH-1, RWPE-

1, DU145, LNCaP, PC3 and 22RV1 analyzed by Western blotting with β-actin as the internal reference. (E) CCK8 cell proliferation curves of FRMD6-overexpressing PC3 cell. Bar graphs are represented as mean ± SD; *P <0.05, **P<0.01, ***P<0.001.

Table S1. Clinicopathological characteristics of PCa patents.

No.	Age	Gender	Primary tumor	Pathological diagnosis	Gleason score	Grade	T stage	PSA (ng/mL)
1	67	M	Yes	adenocarcinoma	5+4=9	5	3	25.82
2	70	M	Yes	adenocarcinoma	4+3=7	3	2	14.61
3	75	M	Yes	adenocarcinoma	3+4=7	2	2	7.03
4	66	M	Yes	adenocarcinoma	3+4=7	2	2	7.85
5	75	M	Yes	adenocarcinoma	3+4=7	2	2	8.84
6	57	M	Yes	adenocarcinoma	3+3=6	1	2	1.62
7	65	M	Yes	adenocarcinoma	3+4=7	2	2	7.46
8	67	M	Yes	adenocarcinoma	3+3=6	1	2	4.93
9	78	M	Yes	adenocarcinoma	4+3=7	3	2	0.67
10	76	M	Yes	adenocarcinoma	3+4=7	2	3	16.28
11	62	M	Yes	adenocarcinoma	4+5=9	5	2	8.13
12	72	M	Yes	adenocarcinoma	5+4=9	5	4	1.1
13	71	M	Yes	adenocarcinoma	3+3=6	1	2	1.54
14	53	M	Yes	adenocarcinoma	3+3=6	1	2	9.69
15	64	M	Yes	adenocarcinoma	3+3=6	1	2	8.31
16	68	M	Yes	adenocarcinoma	3+4=7	2	2	6.68
17	72	M	Yes	adenocarcinoma	4+5=9	5	3	27.08
18	63	M	Yes	adenocarcinoma	4+3=7	3	3	10.34
19	71	M	Yes	adenocarcinoma	3+4=7	2	2	7.31
20	68	M	Yes	adenocarcinoma	3+4=7	2	2	4.61
21	76	M	Yes	adenocarcinoma	4+3=7	3	2	12.78

22	68	M	Yes	adenocarcinoma	3+4=7	2	2	9.66
23	62	M	Yes	adenocarcinoma	3+4=7	2	2	10.3
24	57	M	Yes	adenocarcinoma	4+3=7	3	2	8.2
25	56	M	Yes	adenocarcinoma	3+4=7	2	3	4.86
26	76	M	Yes	adenocarcinoma	4+4=8	4	3	10.45
27	74	M	Yes	adenocarcinoma	3+4=7	2	3	4.64
28	71	M	Yes	adenocarcinoma	4+5=9	5	3	47.58
29	74	M	Yes	adenocarcinoma	3+4=7	2	2	23.36
30	75	M	Yes	adenocarcinoma	4+3=7	3	2	5.3
31	78	M	Yes	adenocarcinoma	3+4=7	2	3	6.63
32	74	M	Yes	adenocarcinoma	3+4=7	2	2	17.51
33	60	M	Yes	adenocarcinoma	3+4=7	2	3	26.32
34	74	M	Yes	adenocarcinoma	3+3=6	1	2	12.9
35	78	M	Yes	adenocarcinoma	3+4=7	2	2	14.61
36	76	M	Yes	adenocarcinoma	4+3=7	3	2	17.52
37	61	M	Yes	adenocarcinoma	4+3=7	3	3	9.57
38	73	M	Yes	adenocarcinoma	3+4=7	2	2	11.57
39	74	M	Yes	adenocarcinoma	4+3=7	3	3	8.09
40	60	M	Yes	adenocarcinoma	5+4=9	5	3	10
41	62	M	Yes	adenocarcinoma	3+4=7	2	2	4.12
42	65	M	Yes	adenocarcinoma	3+4=7	2	2	7.89
43	74	M	Yes	adenocarcinoma	4+3=7	3	2	30.85
44	66	M	Yes	adenocarcinoma	5+4=9	5	2	15.33
45	68	M	Yes	adenocarcinoma	3+3=6	1	2	0.24
46	72	M	Yes	adenocarcinoma	3+4=7	2	3	5.88
47	74	M	Yes	adenocarcinoma	4+3=7	3	2	7.77
48	82	M	Yes	adenocarcinoma	4+3=7	3	3	5.23
49	64	M	Yes	adenocarcinoma	3+4=7	2	2	7.24
50	76	M	Yes	adenocarcinoma	3+4=7	2	2	10.29

51	59	M	Yes	adenocarcinoma	4+3=7	3	3	12.39

Supplementary Table S2. Antibodies used in this study

Antibody name	Source	Catalog	Application (dilution rate)
HNRNPA2B1	Abcam	Ab31645	WB (1:1000), IHC-P (1:500), RIP/CoIP (5ug for each sample)
β-actin	Cell Signaling Technology	#4970	WB (1:1000)
N-cadherin	Cell Signaling Technology	#13116	WB (1:1000)
E-cadherin	Proteintech	20874-1-AP	WB (1:5000)
Snail	Abcam	ab216347	WB (1:1000)
Twist	Abcam	ab175430	WB (1:1000)
Ki67	Cell Signaling Technology	#9449	IHC-P (1:1000)
DGCR8	Proteintech	60084-1-lg	WB (1:1000), RIP/CoIP (5ug for each sample)
METTL3	Proteintech	15073-1-AP	WB (1:2000)
FRMD6	Abcam	Ab110675	WB (1:1000)

Supplementary Table S3. Sequences of sgRNAs (single guide RNAs)

sgRNA	Sequence (5'-3')
SgRNA-HNRNPA2B1-1	Sense: CACCGGCTCGAGAAACAACTCTGCG<br>Antisense: AAACCGCAGAGTTGTTTCTCGAGCC
SgRNA-HNRNPA2B1-2	Sense: CACCGGGAAAGCTTACAGACTGTG<br>Antisense: AAACCACAGTCTGTAAGCTTTCCC

Supplementary Table S4. Oligonucleotides used in this study

Oligonucleotide	Sequence 5'-3'

siMETTL3-1	GCAAGTATGTTCACTATGA
siMETTL3-2	CAAGTATGTTCACTATGAA
miR-93-5p-mimic sense	CAAAGUGCUGUUCGUGCAGGUAG
miR-93-5p-mimic antisense	CUACCUGCACGAACAGCACUUUG
NC-mimic sense	UUUGUACUACACAAAAGUACUG
NC-mimic antisense	CAGUACUUUUGUGUAGUACAAA
hsa-miR-93-5p-inhibitor	CUACCUGCACGAACAGCACUUUG
NC-inhibitor	CAGUACUUUUGUGUAGUACAAA

Supplementary Table S5. PCR primers used in this study

**Primer name**	**Sequence (5'- 3')**
ACTB sense	GGCCGAGGACTTTGATTGCA
ACTB antisense	TGTGTGGACTTGGGAGAGGA
HNRNPA2B1 sense	GGAGCTTTGTCCTAAGTCCTTG
HNRNPA2B1 antisense	ATGTTCCTGCTACCACCAAAG
U6 sense	CTCGCTTCGGCAGCACA
U6 antisense	AACGCTTCACGAATTTGCGT
miR17-5p-RT	GTCGTATCCAGTGCAGGGTCCGAGGTATTCGCACTGGATACGACCTACCT
miR-93-5p-RT	GTCGTATCCAGTGCAGGGTCCGAGGTATTCGCACTGGATACGACCTACCT
miR-3651-RT	GTCGTATCCAGTGCAGGGTCCGAGGTATTCGCACTGGATACGACTCATGT
miR-425-5p-RT	GTCGTATCCAGTGCAGGGTCCGAGGTATTCGCACTGGATACGACTCAACG
miR-20a-5p-RT	GTCGTATCCAGTGCAGGGTCCGAGGTATTCGCACTGGATACGACCTACCT
miR-210-3p-RT	GTCGTATCCAGTGCAGGGTCCGAGGTATTCGCACT

	GGATACGACTCAGCC
miR-17-5p sense	GCGCAAAGTGCTTACAGTGC
miR-17-5p antisense	AGTGCAGGGTCCGAGGTATT
miR-93-5p sense	CGCAAAGTGCTGTTCGTGC
miR-93-5p antisense	AGTGCAGGGTCCGAGGTATT
miR-3651 sense	ATAGCCCGGTCGCTGGT
miR-3651 antisense	AGTGCAGGGTCCGAGGTATT
miR-425-5p sense	GCGAATGACACGATCACTCC
miR-425-5p antisense	AGTGCAGGGTCCGAGGTATT
miR-20a-5p sense	GCGCGTAAAGTGCTTATAGTGC
miR-20a-5p antisense	AGTGCAGGGTCCGAGGTATT
miR-210-3p sense	CGCTGTGCGTGTGACAGC
miR-210-3p antisense	AGTGCAGGGTCCGAGGTATT
pri-miR-93 sense	GTCTTGGACCTCAGTCCTGG
pri-miR-93 antisense	TTGGCAGAGAGAACGTGTCC
pri-miR-425 sense	GCACCTTCAGAATGGAAAGCG
pri-miR-425 antisense	GAGGCGCCGAAAGAGCA
pri-miR-17 sense	AAGTGCTTACAGTGCAGGTAGT
pri-miR-17 antisense	CGAGGCAGCTGTCACCATAA
METTL3 sense	AAGCTGCACTTCAGACGAAT
METTL3 antisense	AAGCTGCACTTCAGACGAAT
FRMD6 sense	TGAAAACCTGCAGCTCAATG
FRMD6 antisense	CTGGGCTGACTTCCAGAGAC
EPHA4 sense	AAAAATGTACTGTGGGGCAGAT
EPHA4 antisense	TCCGTGGAAAGAGCTTTGTAAT
POLR3G sense	CGCAGAACAGGAGGAATATGA
POLR3G antisense	CACTGTCTGCGCCAAAATC
SLC22A23 sense	ACCCCGACGGTGATAAGGTGT
SLC22A23 antisense	TCTGGTTGTGCAGCTCGATGAT

ZNF512B sense	GCTGTGTCCGAAGGAGTTCA
ZNF512B antisense	GTTCTCTGCGTGGGTCTTCA
ENPP5 sense	GCACTAACTCACGCTCATCCT
ENPP5 antisense	CCGTGGTTGCCTAACAGAAAG
MTMR3 sense	AGCAGAGTGGGCTCAGTGTT
MTMR3 antisense	ACTGTCCACGTTTGGTCCTC

Table S6. The expressions of HNRNPA2B1 with clinic pathologic features

**Clinical pathological characters**	**Number**	**HNRNPA2B1 level**		**P value**
		**Low**	**High**	
**Age**				
≤70	25	12	13	0.6763
>70	26	14	13	
**Grade**				
≤3	30	20	10	< 0.01
>3	21	6	15	
**T stage**				
≤2	34	21	13	< 0.05
>2	17	5	12	
